# Fundus Findings in Dengue Fever: A Case Report

**DOI:** 10.4274/tjo.35761

**Published:** 2015-10-05

**Authors:** Berna Şahan, Sinan Tatlıpınar, Deniz Marangoz, Ferda Çiftçi

**Affiliations:** 1 Yeditepe University Faculty of Medicine, Department of Ophthalmology, İstanbul, Turkey

**Keywords:** Dengue fever, Retina, flavivirus

## Abstract

Dengue fever is a flavivirus infection transmitted through infected mosquitoes, and is endemic in Southeast Asia, Central and South America, the Pacific, Africa and the Eastern Mediterranean region. A 41-year-old male patient had visual impairment after travelling to Thailand, which is one of the endemic areas. Cotton wool spots were observed on fundus examination. Fundus fluorescein angiography showed minimal vascular leakage from areas near the cotton wool spots and dot hemorrhages in the macula. Dengue fever should be considered in patients with visual complaints who traveled to endemic areas of dengue fever.

## INTRODUCTION

Dengue fever is an acute viral infection resulting from the transmission of a species of virus in the genus Flavivirus to humans through female mosquitoes of the species Aedes aegypti and Aedes albopticus.^1^ Each year around 50-100 million people are infected with the dengue virus worldwide.^2^ Areas in which it is endemic are South Asia, Central and South America, Africa, and Pacific and Eastern Mediterranean countries.^3^

In dengue fever, a 2 to 7 day incubation period is followed by the sudden onset of symptoms which may include high fever, malaise, headache, retroorbital pain, rhinitis and coughing, nausea and vomiting, and maculopapulary rash. Laboratory tests may reveal thrombocytopenia, leukopenia and hemoconcentration.^4^ Leakage from capillaries and hemorrhage are the basic pathologies of this disease.^5^

Dengue fever patients may have fundus signs such as retinal hemorrhages, macular edema, soft exudates, optic disc edema, vascular sheathing and retinochoroiditis and anterior uveitis.^4,6^

This case report presents the fundus findings of a patient hospitalized for dengue fever while traveling in Thailand.

## CASE REPORT

A 41-year-old male patient presented to our center with complaints of blurred vision. It was learned that the patient had been admitted to the hospital due to a high fever during a trip to Thailand one month earlier. Based on examination results, he had been diagnosed with dengue fever and admitted to the intensive care unit; however, he stated that an ophthalmological examination had not been performed during this period. The patient was not sure exactly when his blurred vision had begun, but reported that the problem was lessening over time. The patient had no history of any systemic diseases, medication use or substance addiction. As it was not possible to obtain the patient’s discharge report documenting the procedures performed abroad, serologic tests and their results from the acute phase could not be analyzed.

**Figure 1:**
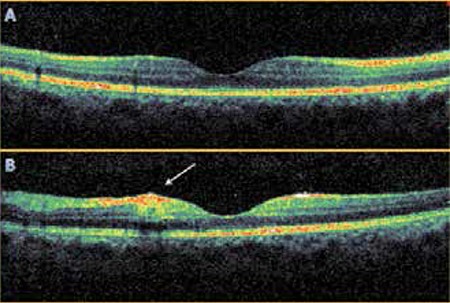
Optic coherence tomography cross-sections showing (A) the right macula is within normal range; (B) in the left macula, there is a field of soft exudates on the nasal side of the fovea with increased reflectivity of the inner retinal layers, and in the foveal area, the outer layers of the retina show more irregularity than in the right eye (arrow)

**Figure 2:**
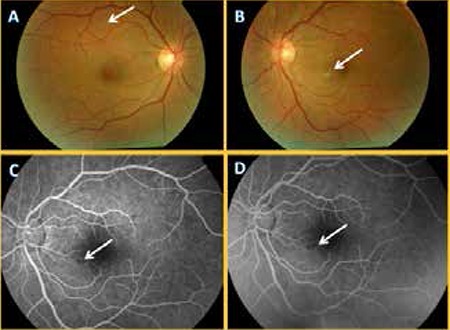
Colored fundus photographs (A, B) showing soft exudates in the right and left eye (arrows); early (C) and late (D) phase fundus fluorescein angiography showing minimal leakage (arrows) from a vasculitic area near the soft exudate field in the inferior nasal left macula

Upon examination in our clinic after returning from Thailand, the patient’s corrected visual acuity was 1.0 right and 0.5 left (Snellen). On slit-lamp examination, the anterior segment appeared normal and no signs of uveitis were detected. Intraocular pressure was 14 mmHg OU. Fundus examination revealed indistinct soft exudates and punctate hemorrhages at the posterior pole which were more pronounced in the left eye. There were no cells in the vitreous. Although there was no increase in retinal thickness in the left macula on optic coherence tomography (OCT), there was increased reflectivity of the inner retinal layers in the area of soft exudate, and the outer retinal layers (ISOS band) in the foveal area were more disorganized compared with the right eye (Figure 1). Fundus fluorescein angiography (FFA) showed indistinct vascular leakage near the area of soft exudate in the left macula (Figure 2). As visual acuity spontaneously returned to normal and no serious ocular involvement was observed in examination, no medication was recommended. Because the patient lived outside the province, he could not come for follow-up.

## DISCUSSION

Dengue fever is the most common viral infection transmitted by a mosquito vector in the world.^7^ Flaviviruses are single-stranded, positive-sense RNA viruses with icosahedral nucleocapsids.^6^ The most common clinical presentation is dengue fever.^6^

Ocular symptoms in dengue fever are self-limiting and usually spontaneously resolve without treatment.^4^ Ophthalmic findings include retinal hemorrhage, macular edema, soft exudates, optic disc edema, vascular sheathing, retinochoroiditis and anterior uveitis.^4,6^ It is believed that the etiopathogenesis of ocular findings related to dengue fever is a mechanism induced more by the immune system than by direct viral infection.^8^ Direct viral infection causes dysfunction and apoptosis of endothelial cells, dendritic cells and monocytes, resulting in cytokine and antibody production. Though not fully proven, it has been proposed that dengue fever maculopathy arises due to the production of antibodies against the retina, retinal pigment epithelium and choroid.^8^ Although controversial, some studies have stated that steroids can be used in cases of maculopathy because it is immune-based.^1,4,7^

OCT, FFA and indocyanine green angiography (ICGA) may be performed in cases of dengue-related maculopathy.^7^ Diffuse retinal thickening and cystoid macular edema can be visualized by OCT.^9^ OCT did not reveal an increase in retinal thickness in our case, which we attribute to the late stage of infection. The lower visual acuity in the left eye was explained by changes in the soft exudate area very close to the fovea.

The most common ocular symptom of dengue fever is blurred vision.^1^ Visual symptoms emerge one week after acute viral infection, which has been attributed to antibody production and immune complex deposition.^1^ Thrombocytopenia, leukopenia and hemoconcentration may occur in dengue fever.^4^ Depending on the course of the disease, increased platelet count has been associated with improvement of visual symptoms.^4,10^

In this case, approximately one month had elapsed between the patient’s hospital stay in Thailand and his presentation to our clinic. During this period, the patient’s visual complaints had decreased with time. There were no important findings during examination other than the soft exudates and punctate hemorrhages at the posterior pole of the fundus, especially in the left eye. This was considered consistent with reports in the literature of ocular findings which spontaneously resolved.

Dengue fever is endemic in South Asian, American, Pacific, African and Eastern Mediterranean countries^3^ Seroepidemiologic studies have indicated that dengue virus may be also present in Turkey.^5^ In patients who develop visual complaints after travel to endemic areas and/or suspicious mosquito bites, ocular involvement related to dengue fever should be considered.
